# Public health preventive measures and child health behaviours during COVID-19: a cohort study

**DOI:** 10.17269/s41997-021-00549-w

**Published:** 2021-07-07

**Authors:** Xuedi Li, Leigh M. Vanderloo, Jonathon L. Maguire, Charles D. G. Keown-Stoneman, Mary Aglipay, Laura N. Anderson, Katherine Tombeau Cost, Alice Charach, Shelley M. Vanderhout, Catherine S. Birken, TARGet Kids! Collaboration, Catherine S. Birken, Jonathon L. Maguire, Ronald Cohn, Eddy Lau, Andreas Laupacis, Patricia C. Parkin, Michael Salter, Shannon Weir-Seeley, Laura N. Anderson, Cornelia M. Borkhoff, Charles Keown-Stoneman, Christine Kowal, Dalah Mason, Murtala Abdurrahman, Kelly Anderson, Gordon Arbess, Jillian Baker, Tony Barozzino, Sylvie Bergeron, Gary Bloch, Joey Bonifacio, Ashna Bowry, Caroline Calpin, Douglas Campbell, Sohail Cheema, Elaine Cheng, Brian Chisamore, Evelyn Constantin, Karoon Danayan, Paul Das, Mary Beth Derocher, Anh Do, Kathleen Doukas, Anne Egger, Allison Farber, Amy Freedman, Sloane Freeman, Sharon Gazeley, Charlie Guiang, Dan Ha, Curtis Handford, Laura Hanson, Leah Harrington, Sheila Jacobson, Lukasz Jagiello, Gwen Jansz, Paul Kadar, Tara Kiran, Holly Knowles, Bruce Kwok, Sheila Lakhoo, Margarita Lam-Antoniades, Eddy Lau, Denis Leduc, Fok-Han Leung, Alan Li, Patricia Li, Jessica Malach, Roy Male, Aleks Meret, Elise Mok, Rosemary Moodie, Katherine Nash, Sharon Naymark, James Owen, Michael Peer, Marty Perlmutar, Navindra Persaud, Andrew Pinto, Michelle Porepa, Vikky Qi, Noor Ramji, Danyaal Raza, Alana Rosenthal, Katherine Rouleau, Caroline Ruderman, Janet Saunderson, Vanna Schiralli, Michael Sgro, Hafiz Shuja, Susan Shepherd, Barbara Smiltnieks, Cinntha Srikanthan, Carolyn Taylor, Stephen Treherne, Suzanne Turner, Fatima Uddin, Meta van den Heuvel, Thea Weisdorf, Peter Wong, John Yaremko, Ethel Ying, Elizabeth Young, Michael Zajdman, Marivic Bustos, Pamela Ruth Flores, Mateenah Jaleel, Tarandeep Malhi, Ataat Malick, Michelle Mitchell, Martin Ogwuru, Frank Ong, Rejina Rajendran, Sharon Thadani, Julia Thompson, Laurie Thompson, Mary Aglipay, Imaan Bayoumi, Sarah Carsley, Katherine Cost, Karen Eny, Laura Kinlin, Jessica Omand, Shelley Vanderhout, Leigh Vanderloo, Christopher Allen, Bryan Boodhoo, Peter Juni, Gurpreet Lakhanpal, Gerald Lebovic, Audra Stitt, Rita Kandel, Michelle Rodrigues

**Affiliations:** 1 Child Health Evaluative Sciences, The Hospital for Sick Children, Toronto, ON Canada; 2 ParticipACTION, Toronto, ON Canada; 3 Department of Pediatrics, Faculty of Medicine, University of Toronto, Toronto, ON Canada; 4 Li Ka Shing Knowledge Institute, St. Michael’s Hospital, Toronto, ON Canada; 5 Dalla Lana School of Public Health, University of Toronto, Toronto, ON Canada; 6 Department of Health Research Methods, Evidence, and Impact, McMaster University, Hamilton, ON Canada; 7 Department of Psychiatry, The Hospital for Sick Children, Toronto, ON Canada; 8 Department of Psychiatry, Faculty of Medicine, University of Toronto, Toronto, ON Canada

**Keywords:** Child health behaviours, COVID-19, Sleep, Screen time, Outdoor, Public health preventive measures, Comportements de santé des enfants, COVID-19, sommeil, temps passé devant l’écran, plein air, mesures préventives de la santé publique

## Abstract

**Objective:**

The primary objective was to determine the association between public health preventive measures and children’s outdoor time, sleep duration, and screen time during COVID-19.

**Methods:**

A cohort study using repeated measures of exposures and outcomes was conducted in healthy children (0 to 10 years) through The Applied Research Group for Kids (TARGet Kids!) COVID-19 Study of Children and Families in Toronto, Canada, between April 14 and July 15, 2020. Parents were asked to complete questionnaires about adherence to public health measures and children’s health behaviours. The primary exposure was the average number of days that children practiced public health preventive measures per week. The three outcomes were children’s outdoor time, total screen time, and sleep duration during COVID-19. Linear mixed-effects models were fitted using repeated measures of primary exposure and outcomes.

**Results:**

This study included 554 observations from 265 children. The mean age of participants was 5.5 years, 47.5% were female and 71.6% had mothers of European ethnicity. Public health preventive measures were associated with shorter outdoor time (−17.2; 95% CI −22.07, −12.40; *p* < 0.001) and longer total screen time (11.3; 95% CI 3.88, 18.79; *p* = 0.003) during COVID-19. The association with outdoor time was stronger in younger children (<5 years), and the associations with total screen time were stronger in females and in older children (≥5 years).

**Conclusion:**

Public health preventive measures during COVID-19 were associated with a negative impact on the health behaviours of Canadian children living in a large metropolitan area.

**Supplementary Information:**

The online version contains supplementary material available at 10.17269/s41997-021-00549-w.

## Résumé

**Objectif:**

L’objectif principal était de déterminer la relation entre les mesures préventives de la santé publique et le temps passé en plein air, la durée du sommeil ainsi que le temps passé devant l’écran par les enfants pendant COVID-19.

**Méthodes:**

Une étude de cohorte utilisant des mesures répétées des expositions et des effets a été menée chez des enfants en bonne santé (0 à 10 ans) par l’entremise de l’Étude COVID-19 sur les Enfants et Familles du Groupe de Recherche Appliquée pour les Enfants (TARGet Kids!) à Toronto, au Canada, entre le 14 avril et le 15 juillet 2020. Les parents ont été invités à remplir des questionnaires sur adhésion aux mesures préventives de la santé publique et les comportements de santé des enfants. La principale exposition était le nombre moyen de jours par semaine durant lesquels les enfants pratiquaient des mesures préventives de la santé publique. Les trois effets étaient le temps passé en plein air par les enfants, le temps total passé devant l’écran et la durée du sommeil pendant le COVID-19. Des modèles linéaires à effets mixtes ont été ajustés en utilisant des mesures répétées d’exposition primaire et des effets.

**Résultats:**

Cette étude comprend 554 observations sur 265 enfants. L’âge moyen des participants était de 5,5 ans, 47,5 % étaient des femmes et 71,6 % avaient des mères d’origine européenne. Les mesures préventives de la santé publique ont été associées à un temps passé en plein air plus court (-17,2 ; IC 95% -22,07, -12,40; *p* < 0,001) et à un temps total devant l’écran plus long (11,3 ; IC 95% 3,88, 18,79; *p* = 0,003) pendant la COVID-19. La relation avec le temps passé en plein air était plus importante chez les jeunes enfants (<5 ans), et les relations avec le temps total passé devant l’écran étaient plus importantes chez les enfants de sexe féminin et les enfants plus âgés (≥5 ans).

**Conclusion:**

Les mesures préventives de la santé publique prises lors de COVID-19 ont été associées à un impact négatif sur les comportements de santé des enfants canadiens vivant dans une grande région métropolitaine.

## Introduction

Healthy movement behaviours are known to improve the physical and psychosocial health of children and youth (Tremblay et al. 2016; Lasselin et al. 2016; World Health Organization 2019). Canada (Tremblay et al. 2016) and the World Health Organization (WHO) (World Health Organization 2019) have recently released 24-h movement behaviour guidelines for children and youth, whereby age-specific recommendations for physical activity, screen time, and sleep are provided (Tremblay et al. 2016). Population-level data suggest less than 15% of Canadian children and youth are meeting these guidelines (Rhodes et al. 2019), limiting the potential for many health benefits. Low levels of outdoor play further exacerbate this issue (Rhodes et al. 2019), once again prohibiting opportunities for children to accrue a healthy balance of movement and sedentary behaviours.

The COVID-19 pandemic resulted in significant changes in the daily lives of individuals, primarily due to the imposed public health restrictions. Canada imposed public health measures that included restrictions requiring physical distancing, social distancing in the form of limited community gatherings and interactions, and limited playground and park use (Government of Canada 2020). For example in March 2020, Ontario mandated closing of schools and non-essential businesses (Detsky and Bogoch 2020; Nielsen 2021). All outdoor recreation amenities including playgrounds, sports fields, and outdoor exercise equipment were shut down (Detsky and Bogoch 2020; Nielsen 2021). Size of social gatherings outside of household was limited to 5 people (Detsky and Bogoch 2020; Nielsen 2021). Restrictions in Ontario started to ease in the summer of 2020 as the new COVID-19 cases declined (Detsky and Bogoch 2020; Nielsen 2021). Beginning on May 11, 2020, children were allowed to walk, hike, and bike in provincial parks (Nielsen 2021). In June 2020, cities in Ontario gradually entered Stage 2 of the province’s recovery plan, allowing for the reopening of outdoor recreational facilities for team sports (with limits on physical distancing) and the size of social gatherings was increased to 10 people (Nielsen 2020). Public health preventive measures including staying home, avoiding visitors at home, and keeping 2 m apart from people outside of household have continued to be widely recommended (Detsky and Bogoch 2020). While implemented to reduce the spread of COVID-19 infection, such measures may have presented additional barriers to maintaining healthy behaviours.

Canadian researchers who examined the impact of the COVID-19 outbreak on children’s and youth’s (5–17 years; *n* = 1472) movement behaviours found that participants engaged in lower levels of physical activity and outdoor play as well as increased levels of sedentary time and sleep in April 2020 (Moore et al. 2020). Another Canadian study showed that children had increased use of screen-based devices and decreased time playing at parks and in public spaces between April and June 2020 (McCormack et al. 2020). Likewise, studies in Italy (Pietrobelli et al. 2020) and China (Xiang et al. 2020) reported similar shifts in behaviour among participants, namely more screen time and shorter physical activity time during COVID-19. However, these studies examined the general impact of the COVID-19 outbreak on children’s health behaviours by comparing the behaviours before and during the outbreak. Little is known about the impact of specific public health preventive measures (i.e., staying at home, limiting visitors at home, avoiding contact with others, keeping distance from others) practiced by children on their health behaviours during COVID-19.

The primary objective of this study was to determine whether the public health preventive measures adopted by children were associated with children’s outdoor time, sleep duration, and screen time during the COVID-19 pandemic. Secondary objectives included determining whether this association differed in males vs. females and also younger vs. older children, and whether adherence to specific public health measures were associated with children’s three health behaviours (outdoor time, sleep duration, and screen time) during COVID-19. We hypothesized that adhering to public health measures was associated with shorter outdoor time, longer screen time, and longer sleep duration in children during COVID-19.

## Methods

### Study design and participants

A cohort study using repeated measures of exposures and outcomes was conducted in healthy children (0 to 10 years) through The Applied Research Group for Kids (TARGet Kids!) COVID-19 Study of Children and Families in Toronto, Canada between April 14 and July 15, 2020 (Carsley et al. 2015). TARGet Kids! is a practice-based research network in Canada, enrolling healthy children, at ages birth to 5 years, from primary health care settings and following them into adolescence (Carsley et al. 2015).

The TARGet Kids! COVID-19 Study of Children and Families aims to describe the impact of the COVID-19 pandemic on children and their parents in the Greater Toronto Area in Canada and to inform prevention efforts against COVID-19 infection for children and parents. Multiple questionnaires were developed for this study and they were administered either weekly, bi-weekly or monthly to participating parents. Families were invited to complete repeated questionnaires either over the telephone or online via REDCap (Harris et al. 2009) about physical and mental health, and health behaviours of children and parents (i.e., outdoor play, sleep, screen time), adherence to public health measures, school, and daycare attendance, and socio-demographic information (i.e., changes in employment, income, subsidies). Informed verbal consent was obtained over the telephone from TARGet Kids! participating families.

### Exposures

The primary exposure in this study was the average number of days reported by parents that children practiced public health preventive measures per week, ranging from 0 to 7 days (Table 1), obtained from the weekly questionnaire. The average was calculated from four individual measures. Secondary exposures were the number of days reported by parents of children and family practicing each of the four individual measures per week: staying at home; limiting the number of visitors at home; avoiding contact with others; and keeping distance from others (Table 1). Exposures greater than 7 days per week were considered implausible and subsequently removed from the sample.

**Table 1:**
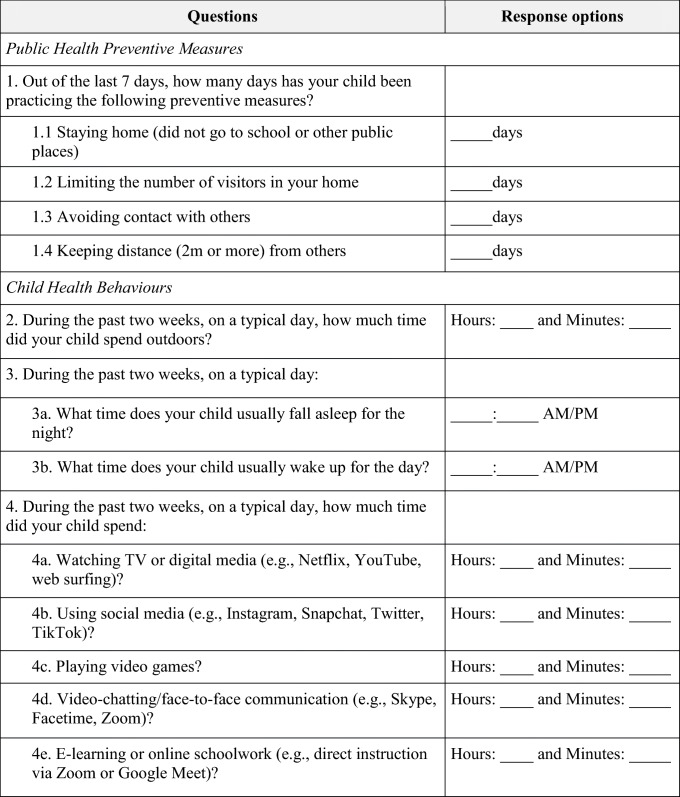
Questions on public health preventive measures and child health behaviour measures from the TARGet Kids! COVID-19 Study of Children and Family questionnaires

### Outcomes

The outcome variables were parent proxy-reported outdoor time, sleep duration, and screen time per day for each child during COVID-19 and were obtained from the study’s bi-weekly questionnaires (Table 1). Sleep duration was the difference between time went to bed (question 3a in Table 1) and time woke up (question 3b in Table 1). Screen time was the sum of question 4a through 4e in Table 1. Separate analyses were performed for video-chatting and e-learning screen time outcomes, as they are pro-social, and may have a positive impact on children during COVID-19. Outdoor time observations were removed if they were greater than or equal to 10 h/day. Sleep duration observations were removed if the time woke up was before midnight on the same day of time went to bed. Screen time observations were removed if one of the subvariables used to compute the overall screen time variable was equal to or exceeded 10 h/day, or if the total screen time was greater than or equal to 12 h/day.

### Covariates

Confounders identified a priori from the literature included child age (Tremblay et al. 2016), child sex (Armstrong et al. 2018; Jones 2017; Statistics Canada, 2019), maternal ethnicity (Armstrong et al. 2018; Jay et al. 2020; Webb Hooper et al. 2020; Love et al. 2019; Anderson et al. 2008; Boxberger and Reimers 2019), self-reported family income (Armstrong et al. 2018; Jay et al. 2020; Webb Hooper et al. 2020; Love et al. 2019; Anderson et al. 2008; Boxberger and Reimers 2019), unemployment due to COVID-19 (Armstrong et al. 2018; Jay et al. 2020; Webb Hooper et al. 2020; Love et al. 2019; Anderson et al. 2008; Boxberger and Reimers 2019), receipt of government subsidies (Armstrong et al. 2018; Jay et al. 2020; Webb Hooper et al. 2020; Love et al. 2019; Anderson et al. 2008; Boxberger and Reimers 2019), calendar date, and living space (Webb Hooper et al. 2020; Statistics Canada 2020; Lambert et al. 2019). Unemployment due to COVID-19 was obtained from the following question: *Have you been unemployed as a result of the COVID-19 pandemic? (Yes/No)*. Receipt of government subsidies was also obtained from the following question: *Have you received government subsidies as a result of the COVID-19 pandemic? (Yes/No)*. Calendar date was the date when the questionnaire containing the exposure variables was completed, and it may capture other time-varying external changes, such as weather, and updates in public health recommendations. We used restricted cubic splines with 5 knots to accommodate various shapes for the association of calendar time in the models. Living space options included apartment or house to account for accessibility of outdoor space. The number of siblings was included as a covariate (Kracht and Sisson 2018). Child age and sex were determined as potential effect modifiers a priori. Child age, unemployment due to COVID-19, receipt of subsidies, calendar date, and living space were collected from the first administered questionnaire. Data for the remaining covariates were collected using a parent-completed, standardized questionnaire adapted from the Canadian Community Health Survey (Government of Canada n.d.).

### Statistical analysis

For our primary analyses, linear mixed-effects models were fitted using repeated measures of primary exposure and outcomes. Since the study included some children from the same family, we included random intercepts for family and subject within family. Separate linear mixed-effects models were fitted for each of the three main outcomes using both unadjusted models and adjusted models with all covariates. Similar unadjusted and adjusted models were used for the secondary analyses which evaluated exposure to each of the four individual public health measures for each of the three outcomes as well as the two pro-social screen time questions. Likelihood ratio tests were used to assess the evidence that child sex or age modified the associations between the exposures and the outcomes. A post hoc exploratory analysis was performed to examine household income as an effect modifier.

Missingness for each covariate was under 15%. Multiple imputation (*n* = 15) was performed using the *mice* package in R to account for bias introduced from missing data (Van Buuren and Groothuis-oudshoorn 2011). All *p*-values were two-tailed and statistical significance was set at α = 0.05. For likelihood ratio tests for interaction, a threshold of *p *< 0.3 was used (Harrell 2015). R version 4.0.2 was used for all analyses (R Core Team 2018 https://www.R-project.org/).

### Ethics approval

This study was approved by the Research Ethics Boards at The Hospital for Sick Children and Unity Health Toronto.

## Results

**Fig. 1:**
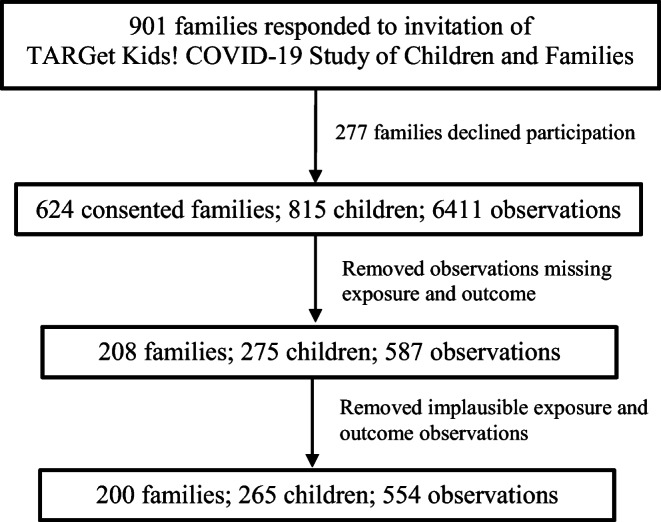
Sample size flow chart

A total of 265 children with 554 observations were included in this study (Fig. 1). Participant characteristics are presented in Table 2. The mean age of participants was 5.5 years, 47.5% were female and 71.6% had mothers of European ethnicity. Of the 265 subjects, 118 (45%) had 1 observation per subject, 147 (55%) had at least 2 observations per subject: of the latter, 56 had 2 observations per subject and 91 had more than 2 observations per subject. The mean follow-up duration for subjects with more than 1 observation (285 follow-ups in total) was 17.1 days. Children in this study came from 199 families: 142 families had 1 child per family, 51 families had 2 children, and 7 families had 3 children.

**Table 2:** Participant characteristics (*N* = 265)

	*N* (Missing)	Mean (*SD*) or *N* (%)
Child age (months) ^a^	265 (0.0%)	66.0 (29.7)
Child sex ^b^	265 (0.0%)	
Female		126 (47.5%)
Male		139 (52.5%)
Maternal ethnicity ^b^	236 (11%)	
European		169 (71.6%)
East/Southeast Asian		29 (12.3%)
South Asian		12 (5.1%)
Black African		3 (1.3%)
Indigenous		0 (0.0%)
Latino		9 (3.8%)
Middle Eastern		1 (0.4%)
Mixed ethnicity		13 (5.5%)
Household income ^b^	242 (8.7%)	
$0 to $39,999		6 (2.5%)
$40,000 to $79,999		23 (9.5%)
$80,000 to $149,999		71 (29.3%)
$150,000 +		142 (58.7%)
Unemployment due to COVID-19 ^a^	264 (0.4%)	
Yes		28 (10.6%)
No		236 (89.4%)
Receipt of subsidies ^a^	264 (0.4%)	
Yes		73 (27.7%)
No		191 (72.3%)
Living space ^a^	231 (12.8%)	
House		201 (87.0%)
Apartment		30 (13.0%)
# of siblings ^b^	244 (8.0%)	0.9 (0.7)
Observations per subject	265 (0.0%)	
1		118 (44.5%)
2		56 (21.1%)
3		49 (18.5%)
4		33 (12.5%)
5		9 (3.4%)
Follow-up duration (days) for subjects with more than 1 observation	285 follow-ups	17.1 (range: 7 to 45)

Notes. ^a^ First-time measure during COVID-19; ^b^ Last measure before COVID-19.

*SD*, standard deviation

Between April 14 and July 15, 2020, the average number of days children practiced staying at home, limiting visitors at home, avoiding contact with others, and keeping distance from others was 5.9, 6.5, 5.8, and 5.9 days per week, respectively (Table 3). The primary exposure, which was the average of the above four measures, was 6.0 days per week.

**Table 3:** Adherence to public health preventive measures and child health behaviours (554 observations, *N* = 265)

	Mean (*SD*)
# of days staying at home per week	5.9 (1.8)
# of days limiting the number of visitors at home per week	6.5 (1.1)
# of days avoiding contact with others per week	5.8 (1.8)
# of days keeping distance (2 m or more) from others per week	5.9 (1.7)
Average of the above 4 measures per week	6.0 (1.2)
Outdoor time per day (min)	185 (103)
Sleep duration per day (min)	638 (60)
Total screen time per day (min)	162 (134)
Video-chatting/face-to-face communication per day (min)	19 (39)
E-learning or online schoolwork per day (min)	18 (44)

Our outcomes were as follows: children’s average outdoor time (185 min/day; 3h05/day); sleep duration (638 min/day; 10h38/day), screen time (162 min/day; 2h42/day). Average video-chatting time was 19 min per day and average e-learning time was 18 min per day.

In the primary analysis (Table 4), it was estimated that for every additional day per week that children adhered to public health preventive measures, outdoor time decreased by 17.2 min per day in the unadjusted model (95% CI: −22.07, −12.40; *p* < 0.001) and decreased by 12.5 min per day in the adjusted model (95% CI: −18.25, −6.79; *p* < 0.001). For every additional day per week that children adhered to public health preventive measures, screen time increased by 11.3 min per day in the unadjusted model (95% CI: 3.88, 18.79; *p* = 0.003). We performed a post hoc analysis adjusting for all covariates, excluding calendar date in the model to explore the role calendar date played in the association, and the resulting effect estimate was similar to the unadjusted model (β=11.30, 95% CI 3.87, 18.73; *p* = 0.003). There was evidence to suggest that age modified the association with total screen time (*p=*0.07), and therefore, the analyses were stratified at 5 years of age based on the age groups of the Canadian 24-Hour Movement Guidelines (Tremblay et al. 2016) (Table 4). There was a stronger positive association in children ≥5 years (β = 17.61; 95% CI: 7.57, 27.66; *p* < 0.001) compared with younger children <5 years (β = 4.50; 95% CI: −5.54, 14.54; *p* = 0.38) in the unadjusted model.

**Table 4:** Number of days per week that children adhered to public health preventive measures (average of four individual measures) and child health behaviours during the COVID-19 pandemic from April 14 to July 15, 2020 (554 observations, *N* = 265)

	Unadjusted			Adjusted		
	β	95% CI	*p*-value	β	95% CI	*p*-value
Outdoor time (min/day)						
# of days children practicing public health preventive measures per week	−17.24	−22.07; −12.40	**<0.001**	−12.52	−18.25; −6.79	**<0.001**
Sleep duration (min/day)						
# of days children practicing public health preventive measures per week	−0.77	−3.95; 2.41	0.63	−0.37	−4.23; 3.48	0.85
Total screen time (min/day)						
Overall* # of days children practicing public health preventive measures per week	11.33	3.88; 18.79	**0.003**	6.44	−2.03; 14.90	0.14
<5 years (*N* = 150, 315 observations)	4.50	−5.54; 14.54	0.38	−0.98	−12.45; 10.49	0.87
≥5 years (*N* = 115, 239 observations)	17.61	7.57; 27.66	**<0.001**	11.30	−1.26; 23.87	0.08

Note: Models were adjusted for child age, child sex, maternal ethnicity, self-reported family income, unemployment due to COVID-19, receipt of government subsidies, calendar date, living space, and number of siblings

*CI*, confidence interval

*There was evidence that age modified the association (*p* = 0.07); therefore, analysis was stratified by age

Bold values denote statistical significance at the *p* < 0.05 level

Results from the secondary analyses of the four individual public health measures and behaviour outcomes are presented in Tables 5, 6, and 7. In instances where significant sex- or age-based interactions were reported (Online Resource 1), analyses were stratified, and results presented accordingly. Each of the four individual public health measures adopted by children was associated with shorter outdoor time in both the unadjusted and adjusted models, except for limiting the number of visitors, with a larger effect size in younger children than in older children (β = −9.94, 95% CI: −17.18; −2.71, *p* = 0.01 in children <5 years). The associations between the four individual measures and screen time are shown in Table 7. Limiting visitors was associated with longer screen time in both the adjusted and unadjusted models, and there was evidence that sex modified the association; therefore, the analyses were stratified by sex (Online Resource 1). There was a stronger positive association in females than in males, and a stronger positive association was found in older children. In the subanalyses evaluating video-chatting and e-learning (Online Resource 2), results modelled closely those for total screen time. Results from the post hoc exploratory analysis showed that household income modified several associations; therefore, the analyses were stratified by household income (Online Resource 3). Children from higher-income families (≥$80,000) had stronger associations with longer total screen time and shorter outdoor time compared with children from lower-income families (<$80,000).

**Table 5:** Four individual public health preventive measures adopted by children and outdoor time during COVID-19 (554 observations, *N* = 265)

Outdoor time (min/day)						
	Unadjusted			Adjusted		
	β	95% CI	*p*-value	β	95% CI	*p*-value
# of days of children practicing staying home per week	−7.28	−10.52; −4.05	**<0.001**	−4.39	−7.91;−0.87	**0.01**
# of days of children practicing limiting the number of visitors at home per week *	−7.91	−13.45; −2.36	**0.01**	−4.24	−9.85; 1.37	0.14
# of days of children practicing avoiding contact with others per week	−11.75	−14.97; −8.54	**<0.001**	−8.63	−12.18; −5.07	**<0.001**
# of days of children practicing keeping distance (≥2 m) from others per week	−9.05	−12.47; −5.63	**<0.001**	−4.77	−8.62; −0.92	**0.02**

Note: Models were adjusted for child age, child sex, maternal ethnicity, self-reported family income, unemployment due to COVID-19, receipt of government subsidies, calendar date, living space, and number of siblings

*There was evidence that age modified the association (*p* = 0.06); therefore, analysis was stratified by age (see Online Resource 1)

Bold values denote statistical significance at the *p* < 0.05 level

**Table 6:** Four individual public health preventive measures adopted by children and sleep duration during COVID-19 (554 observations, *N* = 265)

Sleep duration (min/day)						
	Unadjusted			Adjusted		
	β	95% CI	*p*-value	β	95% CI	*p*-value
# of days of children practicing staying home per week	0.84	−1.24; 2.92	0.43	1.44	−0.91; 3.78	0.23
# of days of children practicing limiting the number of visitors at home per week	−3.13	−6.61; 0.36	0.08	−2.46	−6.18; 1.27	0.20
# of days of children practicing avoiding contact with others per week	−0.63	−2.75; 1.49	0.56	−0.59	−2.99; 1.80	0.63
# of days of children practicing keeping distance (≥2 m) from others per week *	−0.50	−2.69; 1.70	0.66	−0.46	−3.01; 2.10	0.73

Note: Models were adjusted for child age, child sex, maternal ethnicity, self-reported family income, unemployment due to COVID-19, receipt of government subsidies, calendar date, living space, and number of siblings

*There was evidence that age modified the association (*p* = 0.08); therefore, analysis was stratified by age (see Online Resource 1)

**Table 7:** Four individual public health preventive measures adopted by children and screen time duration during COVID-19 (554 observations, *N* = 265)

Total screen time (min/day)						
	Unadjusted			Adjusted		
	β	95% CI	*p*-value	β	95% CI	*p*-value
# of days of children practicing staying home per week	7.21	2.31; 12.12	**0.004**	3.48	−1.72; 8.68	0.19
# of days of children practicing limiting the number of visitors at home per week ^†^	13.90	5.78; 22.01	**<0.001**	9.64	1.53; 17.74	**0.02**
# of days of children practicing avoiding contact with others per week *	4.71	−0.32; 9.73	0.07	2.00	−3.34; 7.33	0.46
# of days of children practicing keeping distance (≥2 m) from others per week	3.06	−2.18; 8.30	0.25	0.10	−5.61; 5.80	0.97

Notes: Models were adjusted for child age, child sex, maternal ethnicity, self-reported family income, unemployment due to COVID-19, receipt of government subsidies, calendar date, living space, and number of siblings

^**†**^There was evidence that sex modified the association (*p* = 0.03); therefore, analysis was stratified by sex (see Online Resource 1)

*There was evidence that age modified the association (*p* = 0.04); therefore, analysis was stratified by age (see Online Resource 1)

Bold values denote statistical significance at the *p* < 0.05 level

## Discussion

Results of this study showed that public health preventive measures practiced by children were associated with a decrease in outdoor time and an increase in screen time during the COVID-19 pandemic between April 14 and July 15, 2020. The findings from this study contribute to the growing evidence demonstrating that the COVID-19 pandemic is associated with unhealthy movement behaviours among Canadian children (Moore et al. 2020; McCormack et al. 2020; Riazi et al. 2021).

Sex-related differences in children’s movement behaviours have been established in the literature (Cumming et al. 2012; Statistics Canada, 2019). The present study found that the associations between public health preventive measures and total screen time were stronger in females compared with their male counterparts. This is comparable with another Canadian study (Moore et al. 2020) which reported that females engaged in more social media use than boys, with both groups reporting an increase in screen use exceeding 6 h a day. Specific to age and the associations with public health measures, increases in total screen time and increased outdoor play periods were linked to older (≥5 years) and younger (<5 years) children, respectively, consistent with pre-pandemic findings in a similar Canadian cohort study (Moore et al. 2020).

The positive association with screen time was more pronounced in older children and in females. This finding may help parents, health care providers, and policy makers address excessive screen time use in females and children greater than 5 years old. Once adjusted for covariates, it was found that the association with screen time was no longer statistically significant. We further explored the relationship between public health measures, screen time, and calendar date (Online Resource 4) and found that both the adherence to public health measures and total screen time decreased over time throughout our analysis and speculate that this may be due to change in weather (more favourable) or quarantine fatigue (Zhao et al. 2020).

The post hoc exploratory analysis showed that the associations with longer screen time and shorter outdoor time were stronger in children from high-income families. Current literature has shown that children from lower-income families tend to engage in higher levels of screen use (Sisson et al. 2009; Carlson et al. 2010; Yang-Huang et al. 2017) and spend more time indoors (Delisle Nyström et al. 2019). In this study, children from families with higher parental income tend to favour participation in unstructured and organized sports (Männikkö et al. 2020) (the main venue in which these children accumulate their physical activity). However, given that participation in sports was ceased and training facilities closed due to COVID-19 restrictions, this unaccounted free time might explain the noted increase in screen time in children from high-income families (i.e., an alt activity to fill this newfound or “excess” free time). Conversely, children from low-income families might not have experienced the same loss in physical activity opportunities as accessibility to facilities and programming is a commonly cited barrier in this group (Chang and Kim 2017). The relationship between income and child health behaviours during COVID-19 requires further assessment.

This study is one of the first investigations exploring the link between adhering to public health recommendations and children’s movement behaviours during the outbreak of the novel COVID-19 virus. The use of the cohort design and repeated measures is a strength of the current study to improve the estimates of the associations. Limitations include the small sample size and the inability to make conclusive causal inferences due to the observational nature of the study design and the potential for unmeasured confounding variables (e.g., additional confounders not controlled for, parenting working in-person or from home, unmeasured health conditions). Selection bias may be introduced by incomplete exposure and outcome data, likely due to requirements for repeated completion of multiple questionnaires. Of note, there was a lower completion rate of the questionnaire that included the outcomes of interest in this study, therefore reducing the available sample size. The participant characteristics in the children included in the analysis (*N*=265) and all children from the 786 consented families (*N*=815), however, were similar (Online Resource 5). Self-reporting bias may also have occurred due to the nature of questionnaires used (parent proxy report). Last, our study is nested within a primary care research network and mainly comprises urban children of European ethnicity with a generally higher socio-economic status; therefore, the findings of this work might not be generalizable to other populations, including low-income populations, and need to be replicated in different contexts.

## Conclusion

Supporting healthy movement behaviours among children is key to establishing healthy trajectories. When developing COVID-19 public health guidelines, public health officials should take into account the negative impact of the guidelines on children’s health, since adhering to these public health measures has had a negative impact on children’s outdoor time and screen time. As Ontario is entering the third wave of COVID-19 and another lockdown is being enforced [at time of writing], we recommend that public health officials and our elected leaders provide clear recommendations for children and families to enable participation in physical activity outdoors and invest in safe outdoor opportunities (with limits such as physical distancing) to promote healthy movement behaviours in children during the COVID-19 pandemic. This is important to consider in child-relevant settings such as community spaces, schools, and public parks and spaces. It is challenging for parents/caregivers to reduce screen time during COVID-19. We suggest parents/caregivers use non-judgemental harm reduction strategies to promote healthful screen use and positive parent–child interaction (Vanderloo et al. 2020; Canadian Centre on Substance Use and Addiction 2008). Future studies are needed to evaluate the longer-term consequences of the pandemic and children’s movement behaviours as the public health guidelines and recommendations evolve during COVID-19.

## Contributions to knowledge

What does this study add to existing knowledge?

- In this cohort study conducted in healthy children during the first wave of COVID-19 in Ontario, we demonstrated that public health preventive measures adopted by children were associated with shorter outdoor time and longer total screen time in Canadian children living in a large metropolitan area during COVID-19.
- The findings from this study contribute to the growing evidence demonstrating that the COVID-19 pandemic is associated with unhealthy movement behaviours among children.

What are the key implications for public health interventions, practice, or policy?

- When developing COVID-19 public health guidelines, public health officials should take into account the negative impact of the guidelines on children’s health, since adhering to these public health measures has had a negative impact on children’s outdoor time and screen time.
- We recommend that public health officials and our elected leaders provide clear recommendations for children and families to enable participation in physical activity outdoors and invest in safe outdoor opportunities (with limits such as physical distancing) to promote healthy movement behaviours in children during the COVID-19 pandemic.

## Supplementary Information

ESM 1 (PDF 216 kb)

ESM 2 (PDF 328 kb)

## Data Availability

Data are available upon request by contacting www.targetkids.ca/contact-us/. The full data are not freely available to respect the confidentiality of our participants, ensure data integrity, and avoid scientific overlap between projects. Once initial contact has been made, we request a short research proposal which will be subject to review by the TARGet Kids! Scientific Committee and approval by institutional IRBs.
