# Supplementary material for: Public health preventive measures and child health behaviours during COVID-19: a cohort study
Source: Can J Public Health. 2021 Jul 7;112(5):831–42. doi: 10.17269/s41997-021-00549-w (PMC8261798; doi:10.17269/s41997-021-00549-w)
Supplement: Supplementary file 1 — (PDF 216 kb) [file 41997_2021_549_MOESM1_ESM.pdf]

Public health preventive measures and child health behaviours during COVID-19: A cohort study

Canadian Journal of Public Health

Xuedi Li, MSc [1*], Leigh M. Vanderloo, PhD [1,2*], Jonathon L. Maguire, MD, MSc, FRCPC [3,4], Charles D.G. Keown-Stoneman, PhD [4,5], Mary Aglipay, MSc [4], Laura N. Anderson, PhD [1,6], Katherine Tombeau Cost, PhD [7], Alice Charach, MD, FRCPC [7,8], Shelley M. Vanderhout, RD, PhD [4], Catherine S. Birken, MD, MSc, FRCPC [1,3], , for the TARGet Kids! Collaboration

[1]Child Health Evaluative Sciences, The Hospital for Sick Children, Toronto, Ontario, Canada
[2]ParticipACTION, Toronto, Ontario, Canada
[3]Department of Pediatrics, Faculty of Medicine, University of Toronto, Toronto, Ontario, Canada
[4]Li Ka Shing Knowledge Institute, St. Michael's Hospital, Toronto, Ontario, Canada
[5]Dalla Lana School of Public Health, University of Toronto, Toronto, Ontario, Canada
[6]Department of Health Research Methods, Evidence, and Impact, McMaster University, Hamilton, Ontario, Canada
[7]Department of Psychiatry, The Hospital for Sick Children, Toronto, Ontario, Canada
[8]Department of Psychiatry, Faculty of Medicine, University of Toronto, Toronto, Ontario, Canada

* Contributed equally as co-first authors

Address correspondence to: Xuedi Li**,** Child Health Evaluative Sciences, Peter Gilgan Centre for Research and Learning, The Hospital for Sick Children, 686 Bay St. Toronto, Ontario, Canada M5G 0A4. Phone: +1 416-813-7654 x 301544 Email: xuedi.li@sickkids.ca

Online Resource 1. Individual public health preventive measures and child health behaviours during COVID-19, stratified by age or sex

**Outdoor time (min/day)**						
**< 5 years**						
N = 150, 315 observations	Unadjusted			Adjusted		
	β	95%CI	*p*-value	β	95%CI	*p*-value
# of days of children practicing limiting # of visitors at home per week	-11.91	-18.73; -5.09	**<0.001**	-9.94	-17.18; -2.71	**0.01**
**≥ 5 years**						
N = 115, 239 observations	Unadjusted			Adjusted		
	β	95%CI	*p*-value	β	95%CI	*p*-value
# of days of children practicing limiting # of visitors at home per week	-4.66	-13.82; 4.51	0.32	-0.43	-9.59; 8.72	0.93
**Sleep duration (min/day)**						
**< 5 years**						
N = 150, 315 observations	Unadjusted			Adjusted		
	β	95%CI	*p*-value	β	95%CI	*p*-value
# of days of children practicing keeping distance from others per week	-2.09	-4.86; 0.69	0.14	-1.77	-4.96; 1.42	0.27
**≥ 5 years**						
N = 115, 239 observations	Unadjusted			Adjusted		
	β	95%CI	*p*-value	β	95%CI	*p*-value
# of days of children practicing keeping distance from others per week	2.70	-0.41; 5.81	0.09	3.05	-0.70; 6.80	0.11
**Total screen time (min/day)**						

**Males**						
N = 139, 298 observations	Unadjusted			Adjusted		
	β	95%CI	*p*-value	β	95%CI	*p*-value
# of days of children practicing limiting # of visitors at home per week	2.80	-8.04; 13.63	0.61	-0.32	-11.29; 10.66	0.95
**Females**						
N = 126, 256 observations	Unadjusted			Adjusted		
	β	95%CI	*p*-value	β	95%CI	*p*-value
# of days of children practicing limiting # of visitors at home per week	30.08	18.23; 41.95	**<0.001**	23.78	11.35; 36.20	**<0.001**
**Total screen time (min/day)**						
**< 5 years**						
N = 150, 315 observations	Unadjusted			Adjusted		
	β	95%CI	*p*-value	β	95%CI	*p*-value
# of days of children practicing avoiding contact with others per week	-1.01	-8.23; 6.21	0.78	-4.22	-12.01; 3.57	0.29
**≥5 years**						
N = 115, 239 observations	Unadjusted			Adjusted		
	β	95%CI	*p*-value	β	95%CI	*p*-value
# of days of children practicing avoiding contact with others per week	9.32	3.00; 15.64	**0.004**	6.06	-1.33; 13.45	0.11

Note: Models were adjusted for child age, child sex, maternal ethnicity, self-reported family income, unemployment due to COVID-19, receipt of government subsidies, calendar date, living space and number of siblings.

Online Resource 2. Number of days per week that children practiced public health preventive measures (average of four individual measures) and video-chatting and e-learning time during COVID-19 pandemic from April 14 to July 15, 2020

**Video-chatting/ face-to-face communication (min/day)**						
N = 265, 554 observations	Unadjusted			Adjusted		
	β	95%CI	*p*-value	β	95%CI	*p*-value
**Overall*** # of days children practicing public health preventive measures per week	3.06	0.33; 5.80	**0.03**	1.34	-1.78; 4.46	0.40
**Males** *N* = 139, 298 observations	3.82	1.40; 6.24	**0.002**	2.44	-0.34; 5.22	0.08
**Females** *N* = 126, 256 observations	5.12	0.27; 9.96	**0.04**	2.79	-3.00; 8.59	0.34
**E-learning or online schoolwork (min/day)**						
N = 265, 554 observations	Unadjusted			Adjusted		
	β	95%CI	*p*-value	β	95%CI	*p*-value
# of days children practicing public health preventive measures per week	3.42	0.08; 6.76	**0.04**	2.01	-1.80; 5.82	0.30

Note: Models were adjusted for child age, child sex, maternal ethnicity, self-reported family income, unemployment due to COVID-19, receipt of government subsidies, calendar date, living space and number of siblings.
* There was evidence that sex modified the association ($p = 0.06$), therefore analysis was stratified by sex

Online Resource 3. Post-hoc analysis: adherence to public health preventive measures and child health behaviours during COVID-19, stratified by household income (*N* = 242, 511 observations)

**Total screen time (min/day)**						
	Unadjusted			Adjusted		
	β	95%CI	*p*-value	95%CI	β	*p*-value
# of days children practicing public health preventive measures per week [a]						
**< $80,000** (*N* = 29, 57 observations)	-14.72	-36.94; 7.50	0.18	-12.09	41.15;16.97	0.40
**≥ $80,000** (*N* = 213, 454 observations)	18.35	9.32; 27.37	**<0.001**	12.64	3.69; 21.58	**0.01**
# of days of children practicing limiting # of visitors at home per week [b]						
**< $80,000** (*N* = 29, 57 observations)	-14.72	-36.94; 7.50	0.18	-12.09	-41.15;16.97	0.40
**≥ $80,000** (*N* = 213, 454 observations)	18.35	9.32; 27.37	**<0.001**	12.64	3.69; 21.58	**0.01**
# of days of children practicing keeping distance from others per week [c]						
**< $80,000** (*N* = 29, 57 observations)	-5.41	-15.20; 4.37	0.27	-10.22	-21.22; 0.78	0.07
**≥ $80,000** (*N* = 213, 454 observations)	6.69	0.59; 12.79	**0.03**	2.58	-4.08; 9.24	0.45
**Outdoor time (min/day)**						
	Unadjusted			Adjusted		
	β	95%CI	β	95%CI	β	95%CI
# of days of children practicing keeping distance from others per week [d]						
**< $80,000** (*N* = 29, 57 observations)	-5.47	-13.22; 2.27	0.16	-6.02	-16.48; 4.44	0.24
**≥ $80,000** (*N* = 213, 454 observations)	-10.20	-14.16; -6.25	**<0.001**	-5.00	-9.47; -0.53	**0.03**

Note: Models were adjusted for child age, child sex, maternal ethnicity, self-reported family income, unemployment due to COVID-19, receipt of government subsidies, calendar date, living space and number of siblings.

There was evidence that income modified the association ([a]p=0.02, [b]p=0.001, [c]p<0.001, [d]p=0.06), therefore analysis was stratified by income

Online Resource 5. Comparison of participant characteristics between participants included in the analysis (N=265) and consented participants (N=815)

	Mean (*SD*) or *N* (%)	
	**N=265**	**N=815**
**Child age (months)** [a]	66.0 (29.7)	62.8 (32.0)
**Child sex** [b]		
Female	126 (47.5%)	394 (47.2%)
Male	139 (52.5%)	340 (52.8%)
**Maternal ethnicity** [b]		
European	169 (71.6%)	401 (64.1%)
East Asian	25 (10.6%)	48 (7.7%)
South and Southeast Asian	16 (6.8%)	76 (12.1%)
Black	3 (1.3%)	22 (3.5%)
Mixed Ethnicity	13 (5.5%)	47 (7.5%)
Other*	10 (4.2%)	32 (5.2%)
**Household income** [b]		
$0 to $39,999	6 (2.5%)	31 (4.6%)
$40,000 to $79,999	23 (9.5%)	76 (11.3%)
$80,000 to $149,999	71 (29.3%)	194 (29.0%)
$150,000 +	142 (58.7%)	369 (55.1%)
**Unemployment due to COVID-19** [a]		
Yes	28 (10.6%)	60 (10.5%)
No	236 (89.4%)	510 (89.5%)
**Receipt of subsidies** [a]		
Yes	73 (27.7%)	155 (27.2%)
No	191 (72.3%)	414 (72.8%)
**Living space** [a]		
House	201 (87.0%)	514 (83.8%)
Apartment	30 (13.0%)	99 (16.2%)

**# of siblings** [b]	0.9 (0.7)	0.8 (0.7)

Notes. [a] First-time measure during COVID-19; [b] Last measure before COVID-19. [*] Others included Arab, Latin American and North American Aboriginal.
SD: Standard Deviation
