# Supplementary material for: Public health preventive measures and child health behaviours during COVID-19: a cohort study
Source: Can J Public Health. 2021 Jul 7;112(5):831–42. doi: 10.17269/s41997-021-00549-w (PMC8261798; doi:10.17269/s41997-021-00549-w)
Supplement: Supplementary file 2 — (PDF 328 kb) [file 41997_2021_549_MOESM2_ESM.pdf]

Public health preventive measures and child health behaviours during COVID-19: A cohort study

Canadian Journal of Public Health

Xuedi Li, MSc [1*], Leigh M. Vanderloo, PhD [1,2*], Jonathon L. Maguire, MD, MSc, FRCPC [3,4], Charles D.G. Keown-Stoneman, PhD [4,5], Mary Aglipay, MSc [4], Laura N. Anderson, PhD [1,6], Katherine Tombeau Cost, PhD [7], Alice Charach, MD, FRCPC [7,8], Shelley M. Vanderhout, RD, PhD [4], Catherine S. Birken, MD, MSc, FRCPC [1,3], , for the TARGet Kids! Collaboration

[1]Child Health Evaluative Sciences, The Hospital for Sick Children, Toronto, Ontario, Canada
[2]ParticipACTION, Toronto, Ontario, Canada
[3]Department of Pediatrics, Faculty of Medicine, University of Toronto, Toronto, Ontario, Canada
[4]Li Ka Shing Knowledge Institute, St. Michael's Hospital, Toronto, Ontario, Canada
[5]Dalla Lana School of Public Health, University of Toronto, Toronto, Ontario, Canada
[6]Department of Health Research Methods, Evidence, and Impact, McMaster University, Hamilton, Ontario, Canada
[7]Department of Psychiatry, The Hospital for Sick Children, Toronto, Ontario, Canada
[8]Department of Psychiatry, Faculty of Medicine, University of Toronto, Toronto, Ontario, Canada

* Contributed equally as co-first authors

Address correspondence to: Xuedi Li, Child Health Evaluative Sciences, Peter Gilgan Centre for Research and Learning, The Hospital for Sick Children, 686 Bay St. Toronto, Ontario, Canada M5G 0A4. Phone: +1 416-813-7654 x 301544 Email: xuedi.li@sickkids.ca

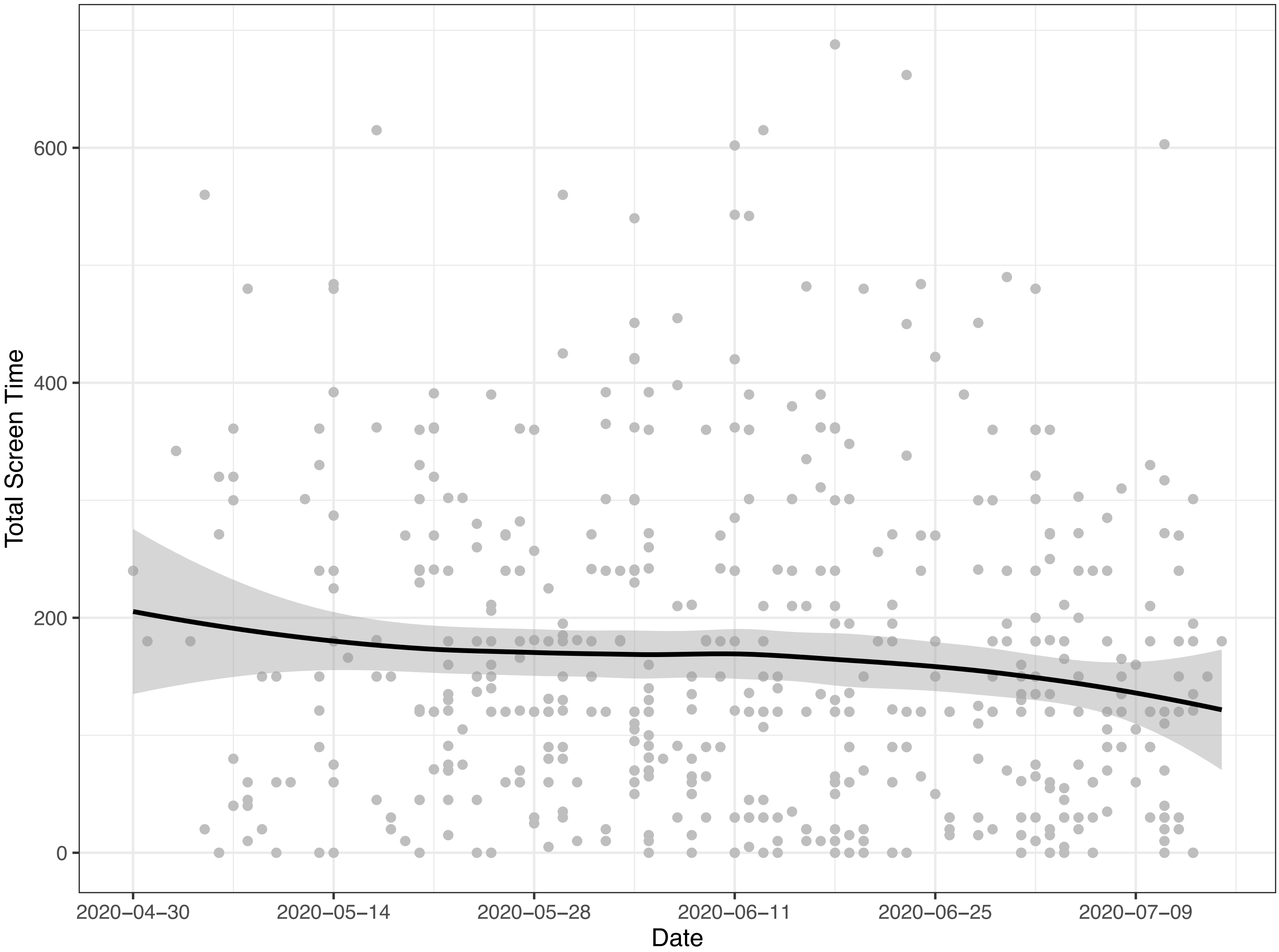

Total Screen Time
600
400
200
0
2020−04−30
2020−05−14
2020−05−28
2020−06−11
2020−06−25
2020−07−09
Date

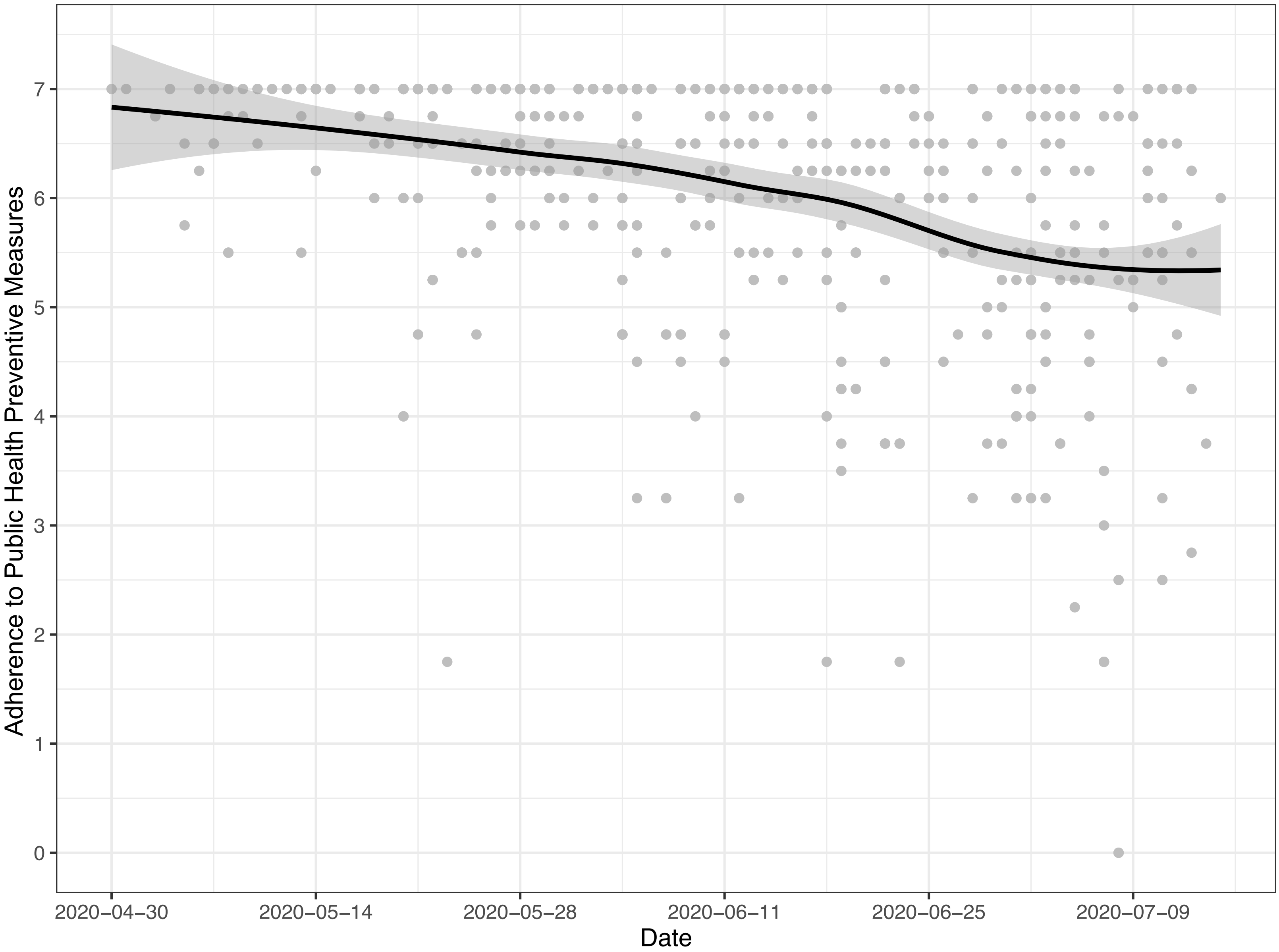

Adherence to Public Health Preventive Measures
0
1
2
3
4
5
6
7
2020−04−30
2020−05−14
2020−05−28
2020−06−11
2020−06−25
2020−07−09
Date

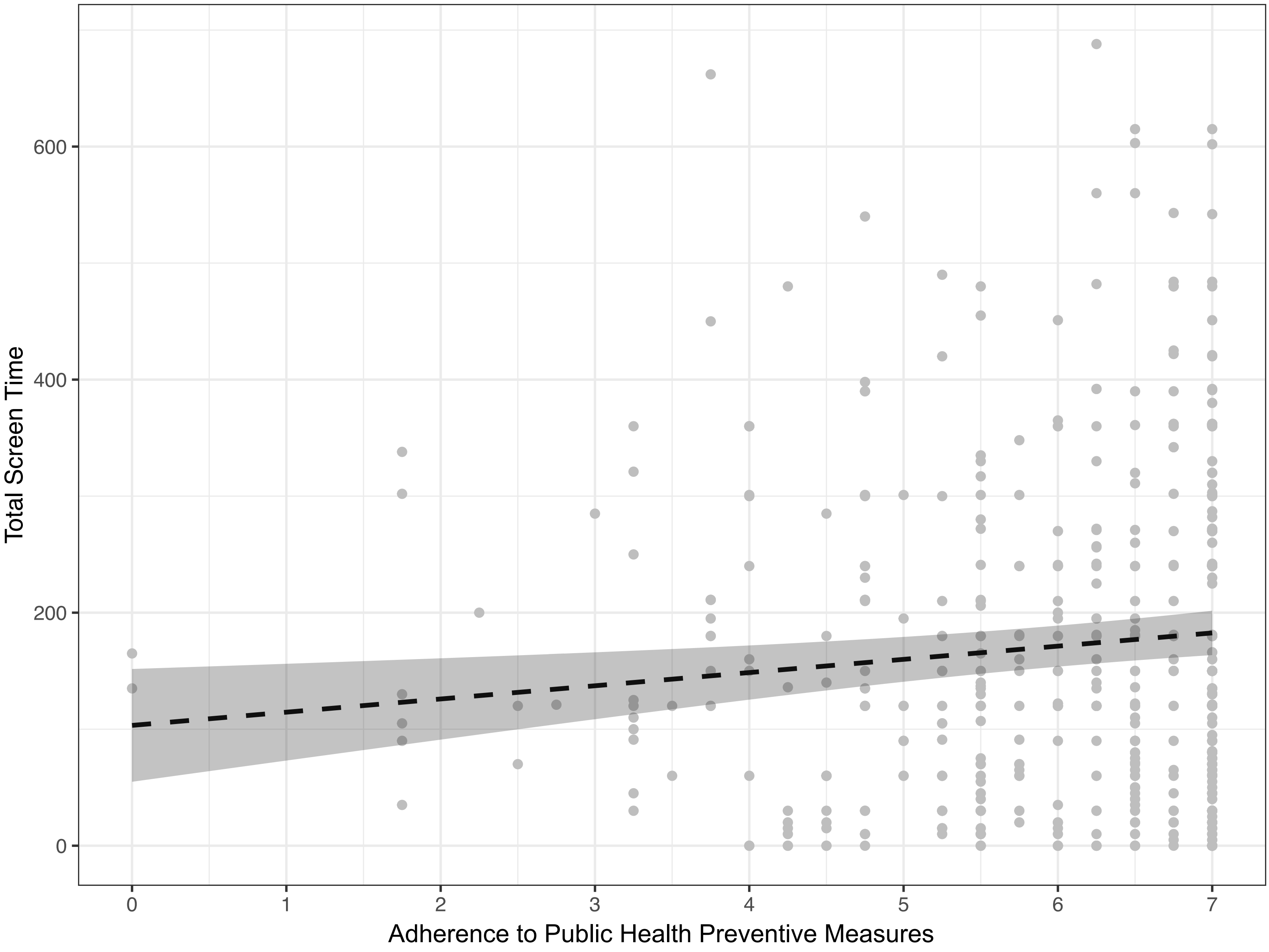

Total Screen Time
600
400
200
0
0
1
2
3
4
5
6
7
Adherence to Public Health Preventive Measures

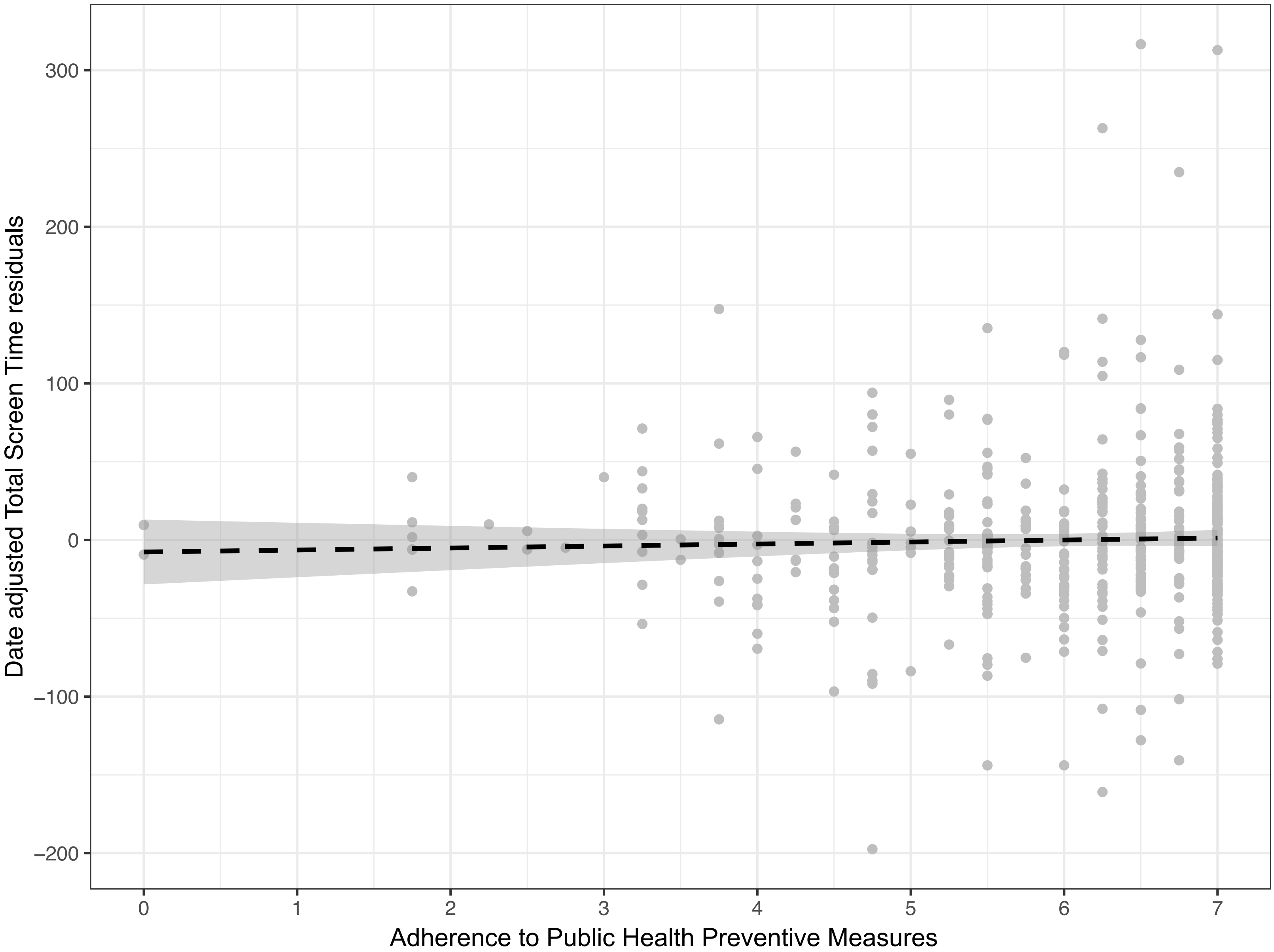

Date adjusted Total Screen Time residuals
300
200
100
0
−100
−200
0
1
2
3
4
5
6
7
Adherence to Public Health Preventive Measures
